# Supplementary material for: Role of UPF1-LIN28A interaction during early differentiation of pluripotent stem cells
Source: Nat Commun. 2024 Jan 2;15:158. doi: 10.1038/s41467-023-44600-5 (PMC10762078; doi:10.1038/s41467-023-44600-5)
Supplement: Supplementary file 1 — Supplementary Information [file 41467_2023_44600_MOESM1_ESM.pdf]

**Supplementary Information**

# Role of UPF1-LIN28A interaction during early differentiation of pluripotent stem cells

Seungwon Jung, Seung Hwan Ko, Narae Ahn, Jinsam Lee, Chang-Hwan Park,*, Jungwook Hwang*

*To whom correspondence should be addressed:

Jungwook Hwang
Tel: +82-2-2220-2427; Fax: +82-2-2220-2422; Email: jwhwang@hanyang.ac.kr

Chang-Hwan Park
Tel: +82-2-2220-0646; Email: chshpark@hanyang.ac.kr

This file contains:

- Supplementary Figure 1-7

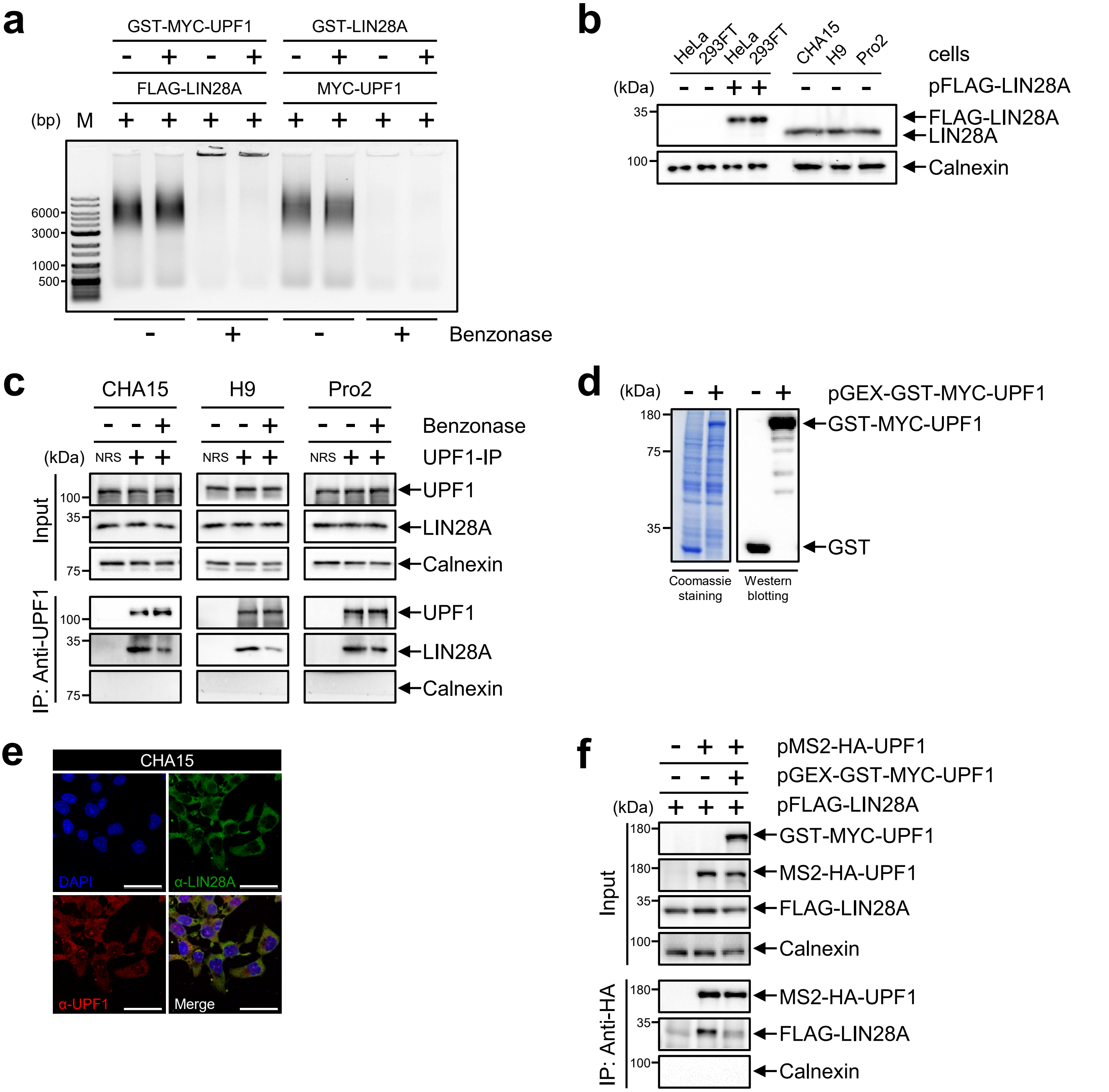


**Supplementary Fig. 1** **a** To determine whether the nuclease (benzonase) completely removed the DNA and RNA, 293T cell lysates that were co-transfected with the indicated plasmid DNA were treated with nuclease and loaded onto an agarose gel. The DNA and RNA were visualised using EtBr staining. **b** The levels of overexpressing LIN28A in HeLa and 293T cells by transfection of FLAG-LIN28A were confirmed by WB to be comparable with the endogenous LIN28A in hPSCs. **c** hPSCs lysates were used for IP using UPF1-antibody in the presence or absence of nuclease (benzonase). **d** Cell lysates expressing GST-MYC-UPF1, or GST before GST pull-down were observed using Coomassie staining and WB. **e** Endogenous UPF1 (red), LIN28A (green), and nucleus (blue, DAPI) in CHA15 cells were visualised using confocal microscopy. Scale bars, 50 μm. **f** 293T cell lysates that were transfected with pFLAG-LIN28A and pMS2-HA-UPF1 or pGEX-GST-MYC-UPF1 were employed for IP using anti-HA antibody. WB was performed to detect coimmunoprecipitated FLAG-LIN28A with HA-UPF1. The minimum number of independent biological replicate experiments was **a**–**f** $n = 3$. The experiments were conducted three times, each iteration producing consistent results.

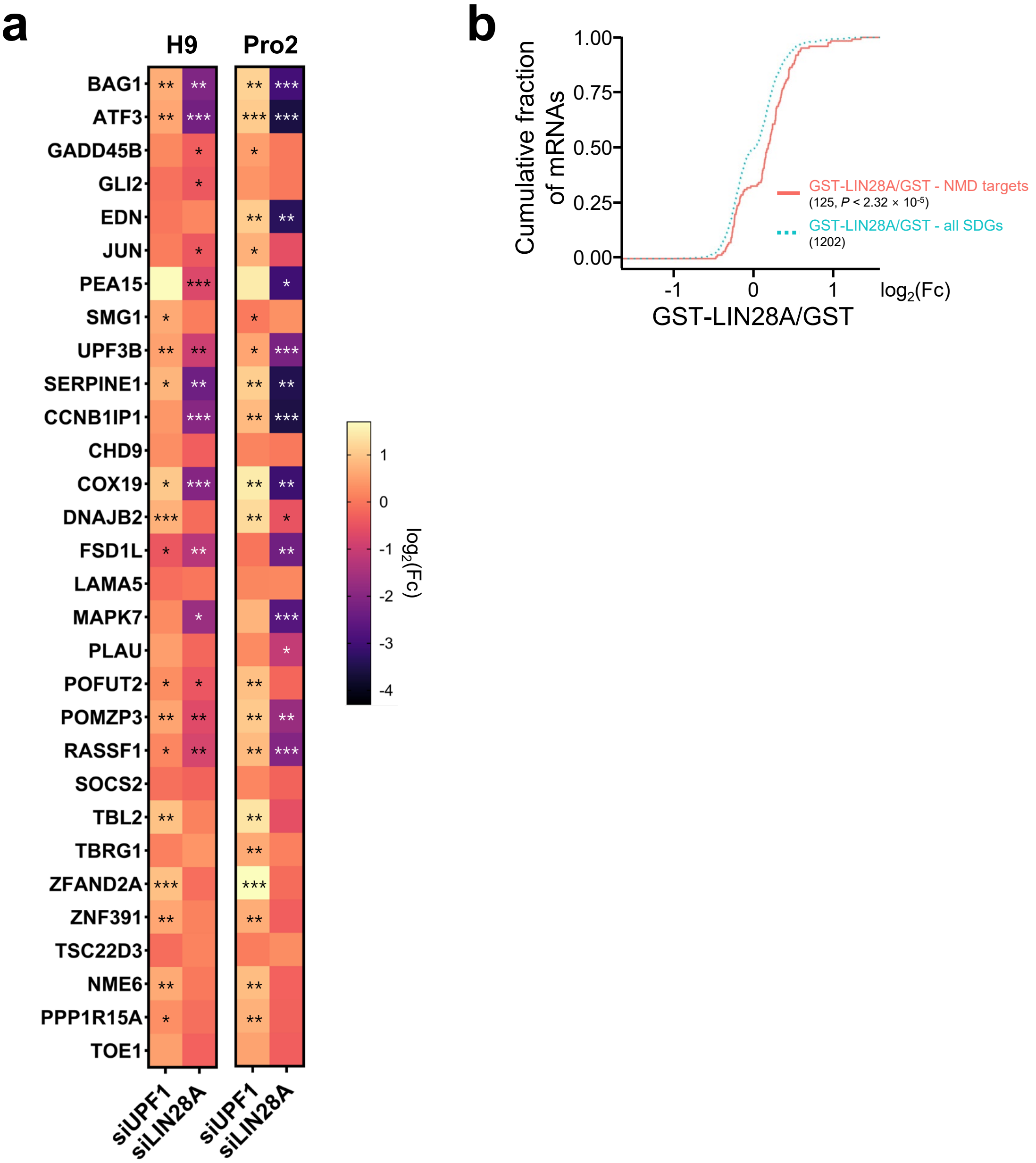


**Supplementary Fig. 2 a** Relative levels of NMD targets by RT-qPCR were presented as heat-maps upon depletion of UPF1 or LIN28A in H9 and Pro2. **b** RNA-seqs using GST-LIN28A- or GST-overexpressing HeLa cells were performed. Cumulative fractions of $\log_2$ changes in the expression of GST-LIN28A against GST were shown as CDF graphs using significantly changed 125 NMD targets and SDGs ($p < 0.05$). mRNA level was normalised to that of *U6* mRNA in **a**. Unpaired Student's t-test was used for **a**. K–S tests were used for **b**. * $p < 0.05$, ** $p < 0.01$, *** $p < 0.001$. Exact p-values were provided in Source data. Data are presented as mean values ± SEM. All statistical test used were two-sided. The minimum number of independent biological replicate experiments was **a** $n = 3$, **b** $n = 2$.

**a**

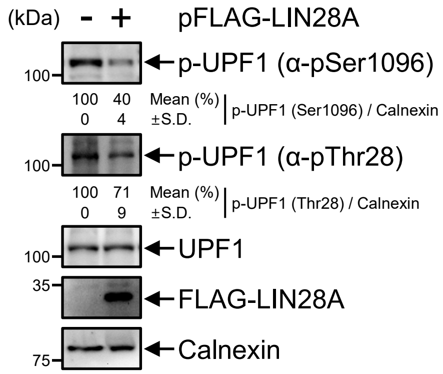


**Supplementary Fig. 3 a** HeLa cells were transfected with pFLAG-LIN28A or pFLAG. WB was performed to detect phosphorylated UPF1 at serine 1096 or threonine 28. Data are presented as mean values ± S.D. All statistical test used were two-sided. The minimum number of independent biological replicate experiments was **a** $n$ = 3. The experiments were conducted three times, each iteration producing consistent results.

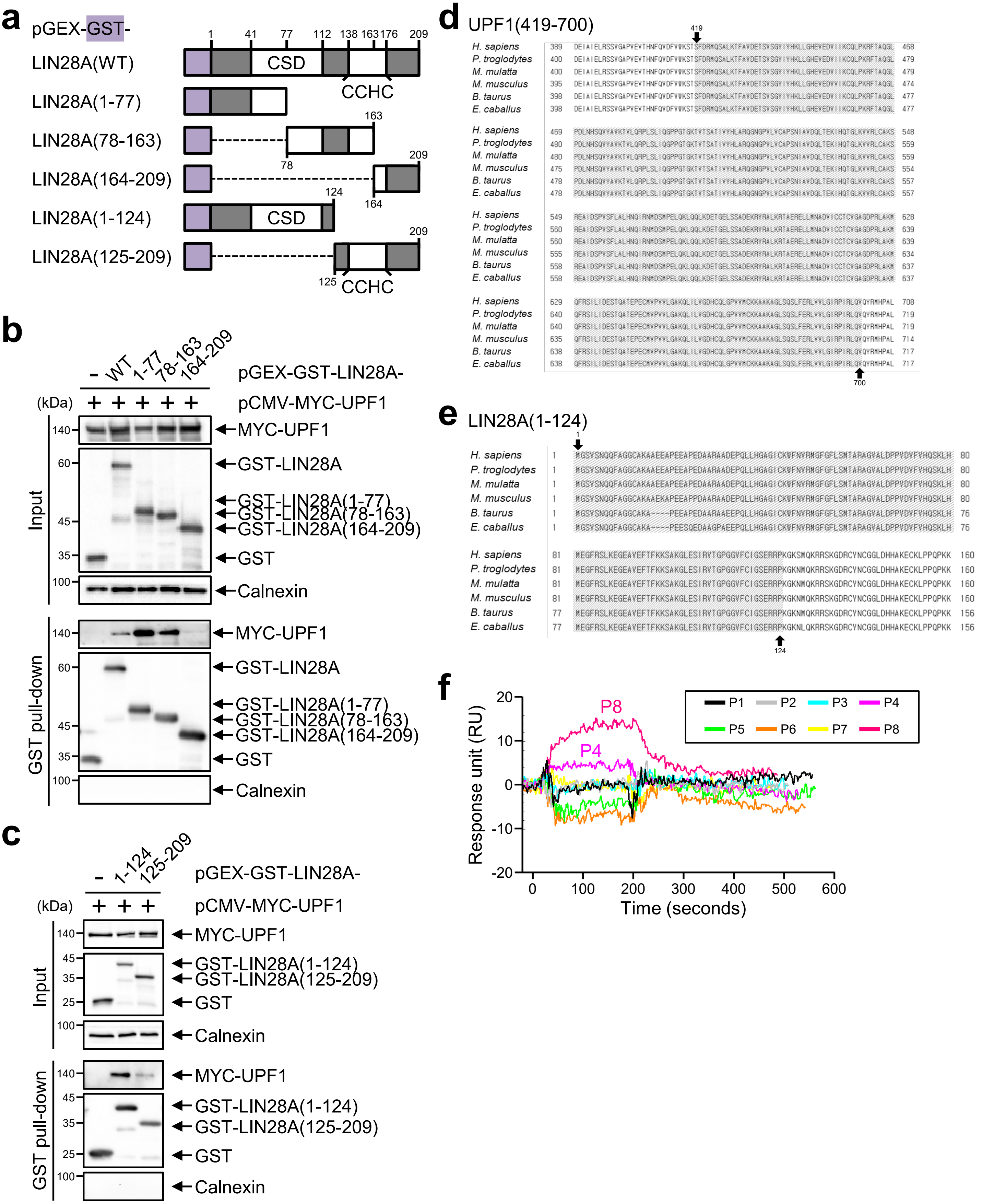
a
pGEX-GST-
LIN28A(WT)
LIN28A(1-77)
LIN28A(78-163)
LIN28A(164-209)
LIN28A(1-124)
LIN28A(125-209)
1 41 77 112 138 163 176 209
CSD
CCHC
b
- WT 1-77 78-163 164-209
pGEX-GST-LIN28A-
(kDa)
pCMV-MYC-UPF1
Input
GST pull-down
140
60
45
35
100
MYC-UPF1
GST-LIN28A
GST-LIN28A(1-77)
GST-LIN28A(78-163)
GST-LIN28A(164-209)
GST
Calnexin
c
- 1-124 125-209
GST-LIN28A(1-124)
GST-LIN28A(125-209)
25
d
UPF1(419-700)
H. sapiens
P. troglodytes
M. mulatta
M. musculus
B. taurus
E. caballus
419
700
e
LIN28A(1-124)
124
f
Response unit (RU)
Time (seconds)
P1 P2 P3 P4
P5 P6 P7 P8
P8
P4
20
10
0
-10
-20
0 100 200 300 400 500 600

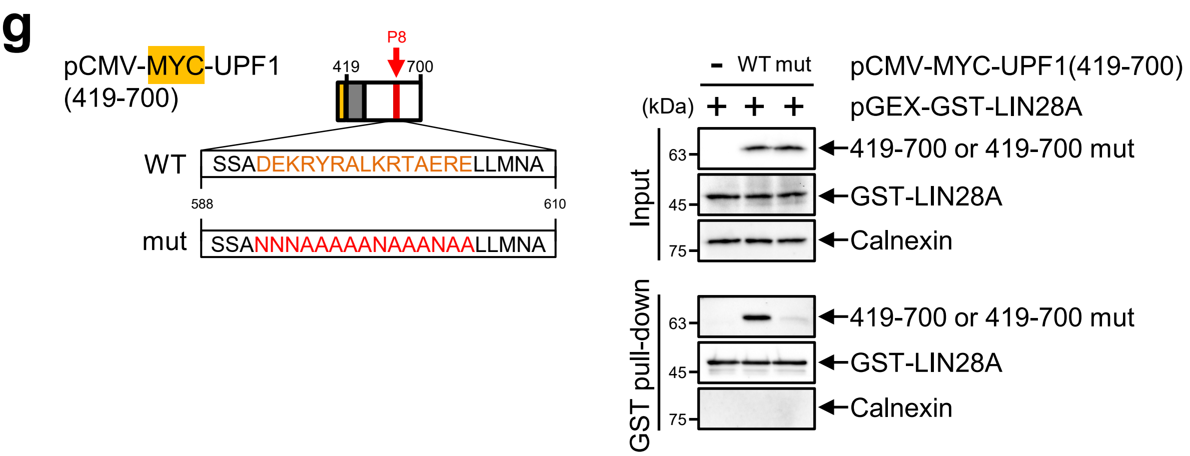


**Supplementary Fig. 4 a** Schematic representation of GST-tagged LIN28A variants. LIN28A is composed of two domains: cold shock domain (CSD) and Cys-Cys-His-Cys (CCHC)[1]. **b** 293T cell lysates that were transfected with pGEX-GST-LIN28A variants (WT, 1-77, 78-163, and 164-209) and pCMV-MYC-UPF1 were employed for GST pull-down. WB was performed to evaluate the expression levels of LIN28A variants and eluates. **c** Same as in (**b**); however, 293T cell lysates were transfected with pGEX-GST-LIN28A variants (1-124 and 125-209). **d–e** Conserved amino acid sequences of putative interacting domain in UPF1 (**d**) and LIN28A (**e**). **f** SPR analysis using the eight peptide analytes as depicted in Fig. 4c, where His-LIN28A protein was used as the ligand. **g** Schematic representation of MYC-UPF1(419-700) and mutation (mut). The fifteen amino acids (red) in the P8 region were substituted with asparagine (N) or alanine (A). 293T cell lysates that were transfected with pGEX-GST-LIN28A and pCMV-MYC-UPF1(419-700) variants (WT or mut) were used for GST pull-down in the presence of nuclease. WB was performed to evaluate the expression levels of UPF1(419-700) variants and eluates. The minimum number of independent biological replicate experiments was **b**, **c**, and **g** n = 3. The experiments were conducted three times, each iteration producing consistent results.

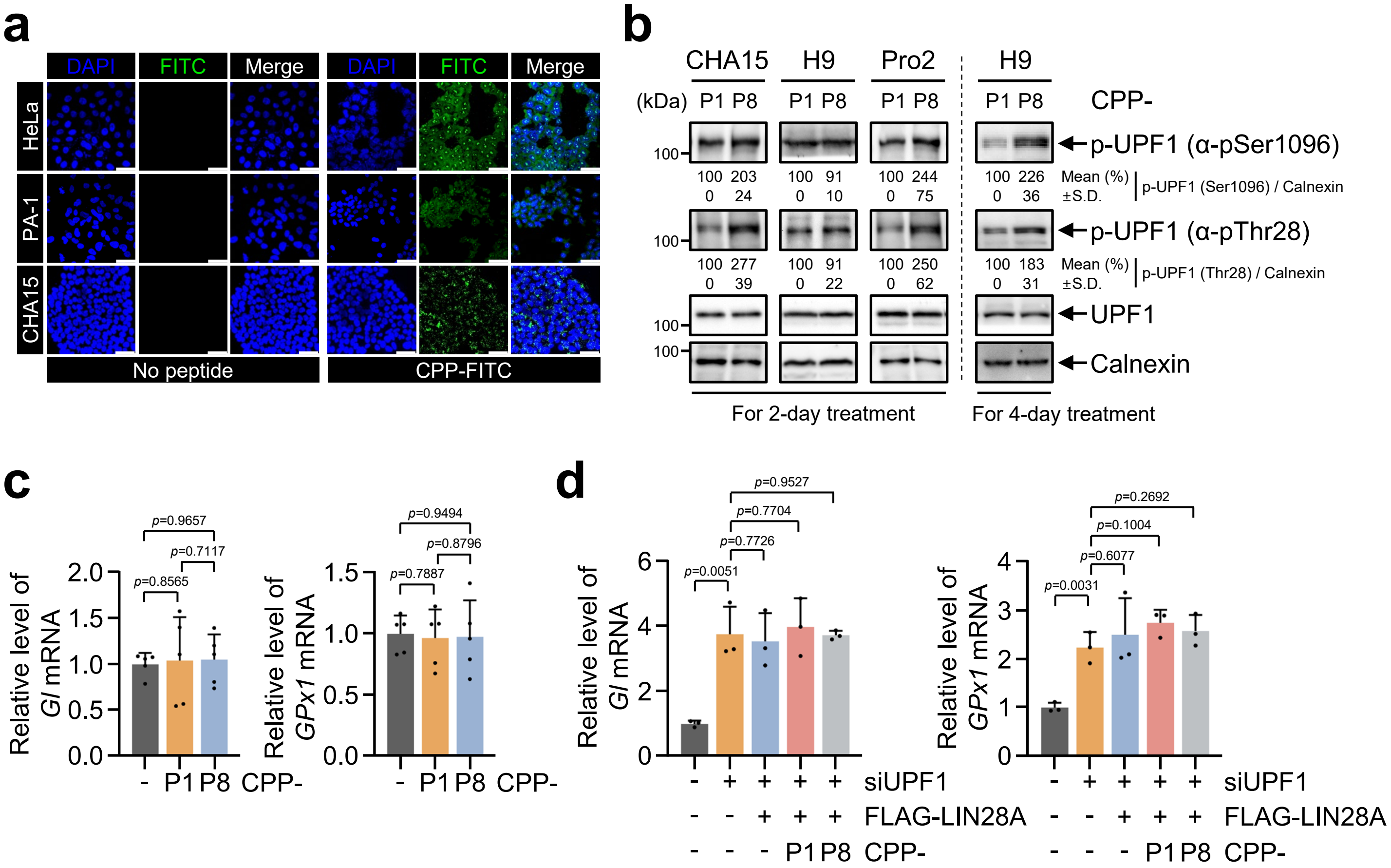


**Supplementary Fig. 5 a** HeLa, PA-1, and CHA15 were treated with CPP-FITC. FITC and DAPI (nucleus) were visualised using confocal microscopy. Scale bars, 50 μm. **b** hPSCs lysates that were treated with CPP-P1 or -P8 were used for WB to detect the levels of p-UPF1. **c** HeLa cells where LIN28A was not expressed were transfected with NMD reporter plasmids and incubated with 2 μM of the indicated CPP-conjugated peptides. RT-qPCR was performed to measure NMD efficiency. **d** Same as (**c**); however, the UPF1-depleted condition was established by siRNA in the presence of FLAG-LIN28A in HeLa cells. mRNA level was normalised to that of *MUP* mRNA in (**c** and **d**). Unpaired Student's t-test was used. Data are presented as mean values ± S.D (**b**) and mean values ± SEM (**c** and **d**). All statistical test used were two-sided. The minimum number of independent biological replicate experiments was **a**, **b**, and **d** $n = 3$, **c** $n = 5$. The experiments were conducted three times, each iteration producing consistent results.

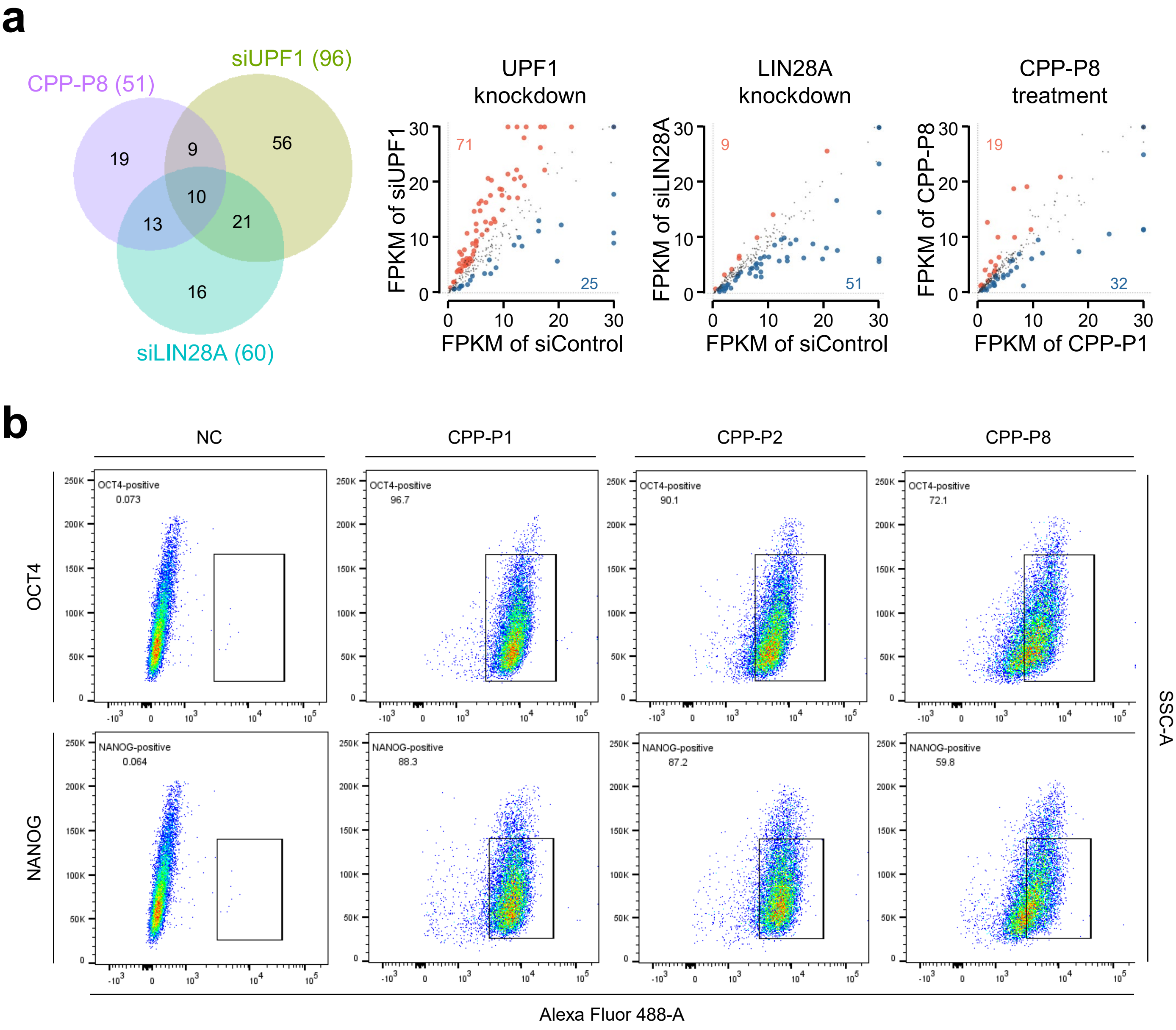
a
CPP-P8 (51)
siUPF1 (96)
siLIN28A (60)
19
9
56
10
13
21
16
UPF1 knockdown
LIN28A knockdown
CPP-P8 treatment
FPKM of siUPF1
FPKM of siLIN28A
FPKM of CPP-P8
FPKM of siControl
FPKM of siControl
FPKM of CPP-P1
71
25
9
51
19
32
b
NC
CPP-P1
CPP-P2
CPP-P8
OCT4
NANOG
OCT4-positive
0.073
96.7
90.1
72.1
NANOG-positive
0.064
88.3
87.2
59.8
SSC-A
Alexa Fluor 488-A

**c**

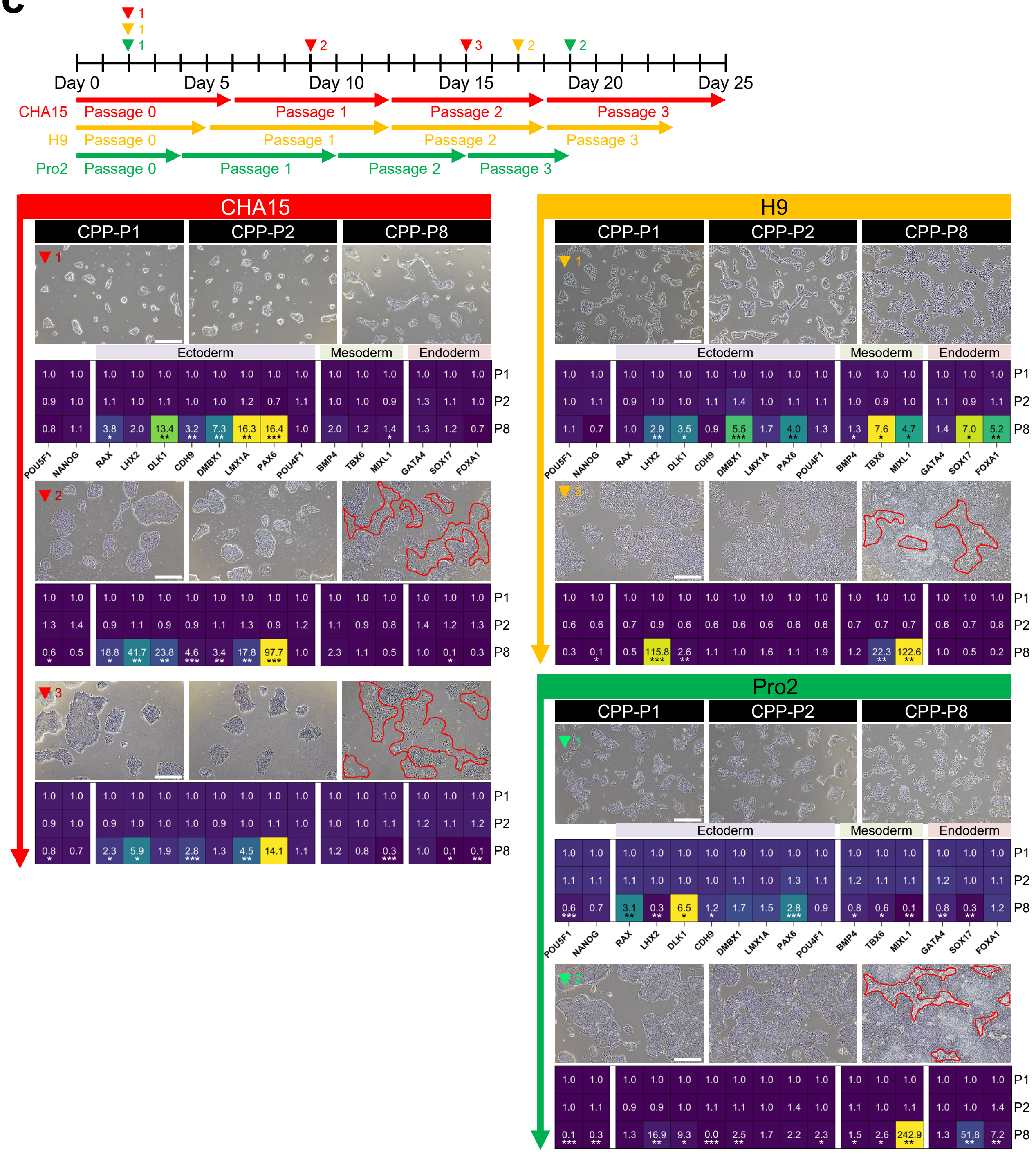
Day 0 Day 5 Day 10 Day 15 Day 20 Day 25
CHA15 Passage 0 Passage 1 Passage 2 Passage 3
H9 Passage 0 Passage 1 Passage 2 Passage 3
Pro2 Passage 0 Passage 1 Passage 2 Passage 3
CHA15
H9
Pro2
CPP-P1 CPP-P2 CPP-P8
Ectoderm Mesoderm Endoderm
POU5F1 NANOG RAX LHX2 DLK1 CDH9 DMBX1 LMX1A PAX6 POU4F1 BMP4 TBX6 MIXL1 GATA4 SOX17 FOXA1
CHA15 1
P1 1.0 1.0 1.0 1.0 1.0 1.0 1.0 1.0 1.0 1.0 1.0 1.0 1.0 1.0 1.0 1.0
P2 0.9 1.0 1.1 1.0 1.1 1.0 1.0 1.2 0.7 1.1 1.0 1.0 0.9 1.3 1.1 1.0
P8 0.8 1.1 3.8 2.0 13.4 3.2 7.3 16.3 16.4 1.0 2.0 1.2 1.4 1.3 1.2 0.7
CHA15 2
P1 1.0 1.0 1.0 1.0 1.0 1.0 1.0 1.0 1.0 1.0 1.0 1.0 1.0 1.0 1.0 1.0
P2 1.3 1.4 0.9 1.1 0.9 0.9 1.1 1.3 0.9 1.2 1.1 0.9 0.8 1.4 1.2 1.3
P8 0.6 0.5 18.8 41.7 23.8 4.6 3.4 17.8 97.7 1.0 2.3 1.1 0.5 1.0 0.1 0.3
CHA15 3
P1 1.0 1.0 1.0 1.0 1.0 1.0 1.0 1.0 1.0 1.0 1.0 1.0 1.0 1.0 1.0 1.0
P2 0.9 1.0 0.9 1.0 1.0 1.0 0.9 1.0 1.1 1.0 1.0 1.0 1.1 1.2 1.1 1.2
P8 0.8 0.7 2.3 5.9 1.9 2.8 1.3 4.5 14.1 1.1 1.2 0.8 0.3 1.0 0.1 0.1
H9 1
P1 1.0 1.0 1.0 1.0 1.0 1.0 1.0 1.0 1.0 1.0 1.0 1.0 1.0 1.0 1.0 1.0
P2 1.0 1.1 0.9 1.0 1.0 1.1 1.4 1.0 1.1 1.1 1.0 0.9 1.0 1.1 0.9 1.1
P8 1.1 0.7 1.0 2.9 3.5 0.9 5.5 1.7 4.0 1.3 1.3 7.6 4.7 1.4 7.0 5.2
H9 2
P1 1.0 1.0 1.0 1.0 1.0 1.0 1.0 1.0 1.0 1.0 1.0 1.0 1.0 1.0 1.0 1.0
P2 0.6 0.6 0.7 0.9 0.6 0.6 0.6 0.6 0.6 0.6 0.7 0.7 0.7 0.6 0.7 0.8
P8 0.3 0.1 0.5 115.8 2.6 1.1 1.0 1.6 1.1 1.9 1.2 22.3 122.6 1.0 0.5 0.2
Pro2 1
P1 1.0 1.0 1.0 1.0 1.0 1.0 1.0 1.0 1.0 1.0 1.0 1.0 1.0 1.0 1.0 1.0
P2 1.1 1.1 1.1 1.0 1.0 1.0 1.1 1.0 1.3 1.1 1.2 1.1 1.1 1.2 1.0 1.1
P8 0.6 0.7 3.1 0.3 6.5 1.2 1.7 1.5 2.8 0.9 0.8 0.6 0.1 0.8 0.3 1.2
Pro2 2
P1 1.0 1.0 1.0 1.0 1.0 1.0 1.0 1.0 1.0 1.0 1.0 1.0 1.0 1.0 1.0 1.0
P2 1.0 1.1 0.9 0.9 1.0 1.1 1.1 1.0 1.4 1.0 1.1 1.0 1.1 1.0 1.0 1.4
P8 0.1 0.3 1.3 16.9 9.3 0.0 2.5 1.7 2.2 2.3 1.5 2.6 242.9 1.3 51.8 7.2

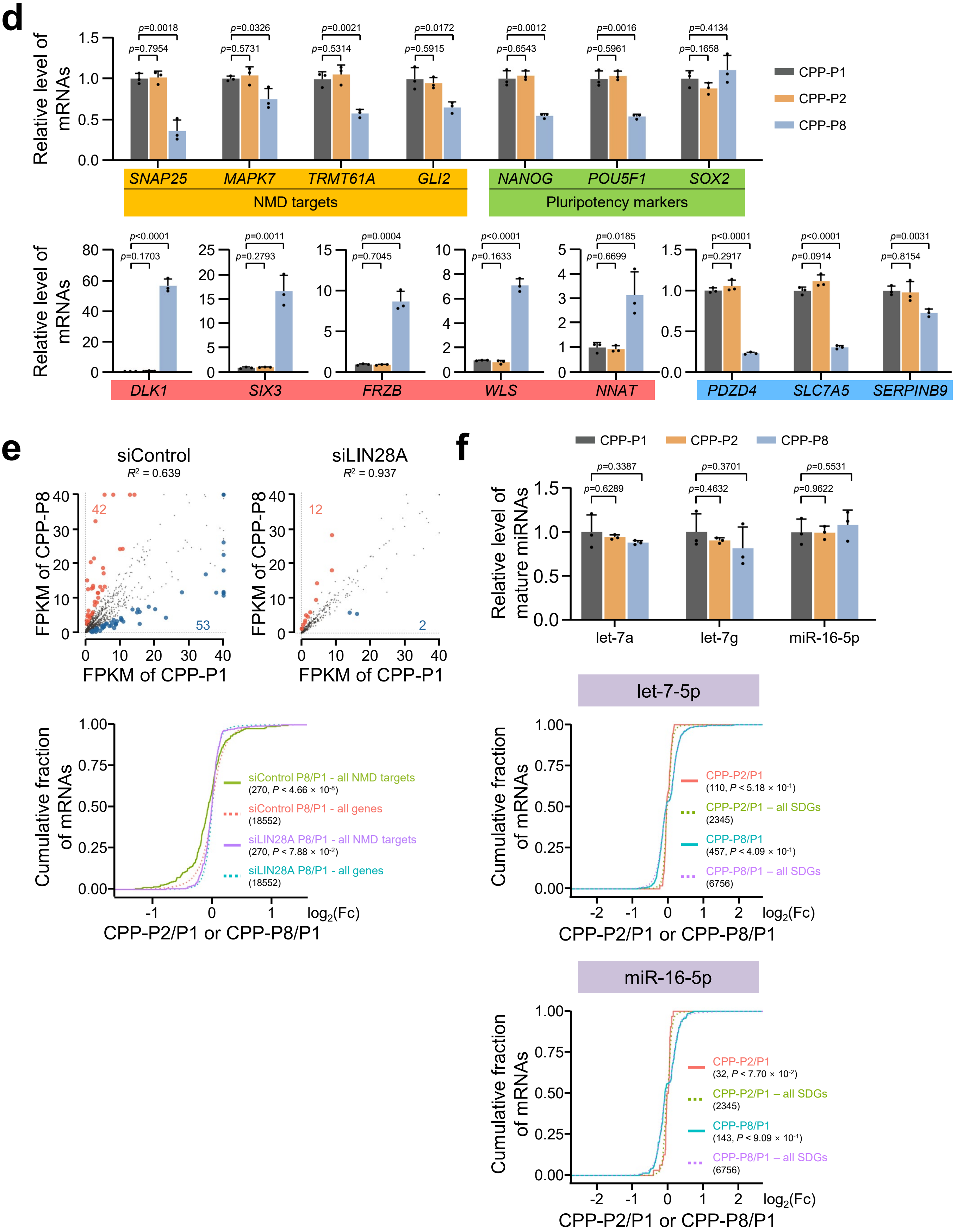

d
Relative level of mRNAs
CPP-P1
CPP-P2
CPP-P8
SNAP25
MAPK7
TRMT61A
GLI2
NANOG
POU5F1
SOX2
NMD targets
Pluripotency markers
DLK1
SIX3
FRZB
WLS
NNAT
PDZD4
SLC7A5
SERPINB9
e
siControl
$R^2$ = 0.639
siLIN28A
$R^2$ = 0.937
FPKM of CPP-P8
FPKM of CPP-P1
Cumulative fraction of mRNAs
siControl P8/P1 - all NMD targets
(270, $P < 4.66 \times 10^{-8}$)
siControl P8/P1 - all genes
(18552)
siLIN28A P8/P1 - all NMD targets
(270, $P < 7.88 \times 10^{-2}$)
siLIN28A P8/P1 - all genes
(18552)
$\log_2$(Fc)
CPP-P2/P1 or CPP-P8/P1
f
Relative level of mature miRNAs
let-7a
let-7g
miR-16-5p
let-7-5p
CPP-P2/P1
(110, $P < 5.18 \times 10^{-1}$)
CPP-P2/P1 – all SDGs
(2345)
CPP-P8/P1
(457, $P < 4.09 \times 10^{-1}$)
CPP-P8/P1 – all SDGs
(6756)
miR-16-5p
CPP-P2/P1
(32, $P < 7.70 \times 10^{-2}$)
CPP-P2/P1 – all SDGs
(2345)
CPP-P8/P1
(143, $P < 9.09 \times 10^{-1}$)
CPP-P8/P1 – all SDGs
(6756)

**Supplementary Fig. 6 a** Scatter plots and Venn diagram shows the number of NMD targets that were significantly changed over 1.2-fold upon UPF1-depletion, LIN28A-depletion, or CPP-P8-treatment in CHA15 cells. **b** (Corresponding to Fig. 6c) Flow cytometry analysis was performed to determine the levels of OCT4 and NANOG in CPP-P1, -P2, or -P8-treated CHA15 cells. The primary antibody that detected the cellular proteins was labelled with the Alexa Fluor 488-conjugated antibody. The relative positive cells were indicated. **c** The schematic representation shows proliferation and passages day point of hPSCs. hPSCs that were treated with CPP-P1, -P2, or -P8 were expanded for the indicated day point with passages. Cell morphology was visualised by phase contrast microscopy. The red boundary indicated the spontaneously differentiated hPSCs. RT-qPCR was performed to measure the relative levels of each lineage marker. Scale bars, 100 μm. **d** (Corresponding to Fig. 6d) RT-qPCR was performed to evaluate the level of indicated transcripts upon the indicated peptide treatment in CHA15 cells. **e** Transcriptome analysis was performed using significantly changed transcripts ($p < 0.05$) from siLIN28A or siControl in the indicated CPP-peptide treated CHA15 cells. Scatter plots and CDFs employed SDGs over a 2.0-fold change (upper panel) and significantly changed the NMD targets (bottom panel), respectively. **f** Levels of mature endogenous miRNAs in CHA15 cells, which were regulated by LIN28A, were assessed using RT-qPCR. Cumulative fraction of $\log_2$ fold change in the expression of genes regulated by let-7-5p and miR-16-5p was shown as CDF graphs. The relative level of mRNA was normalised to that of *U6* snRNA in (**c**, **d**, and **f**). K–S tests were used for **e** and **f**. Unpaired Student's t-test was used for **c**, **d**, and **f**. * $p < 0.05$, ** $p < 0.01$, *** $p < 0.001$. Exact *p*-values were provided in Source data. Data are presented as mean values ± SEM. All statistical test used were two-sided. The minimum number of independent biological replicate experiments was **a** and **e** $n = 2$, **b**–**d** and **f** $n = 3$. The experiments were conducted three times, each iteration producing consistent results.

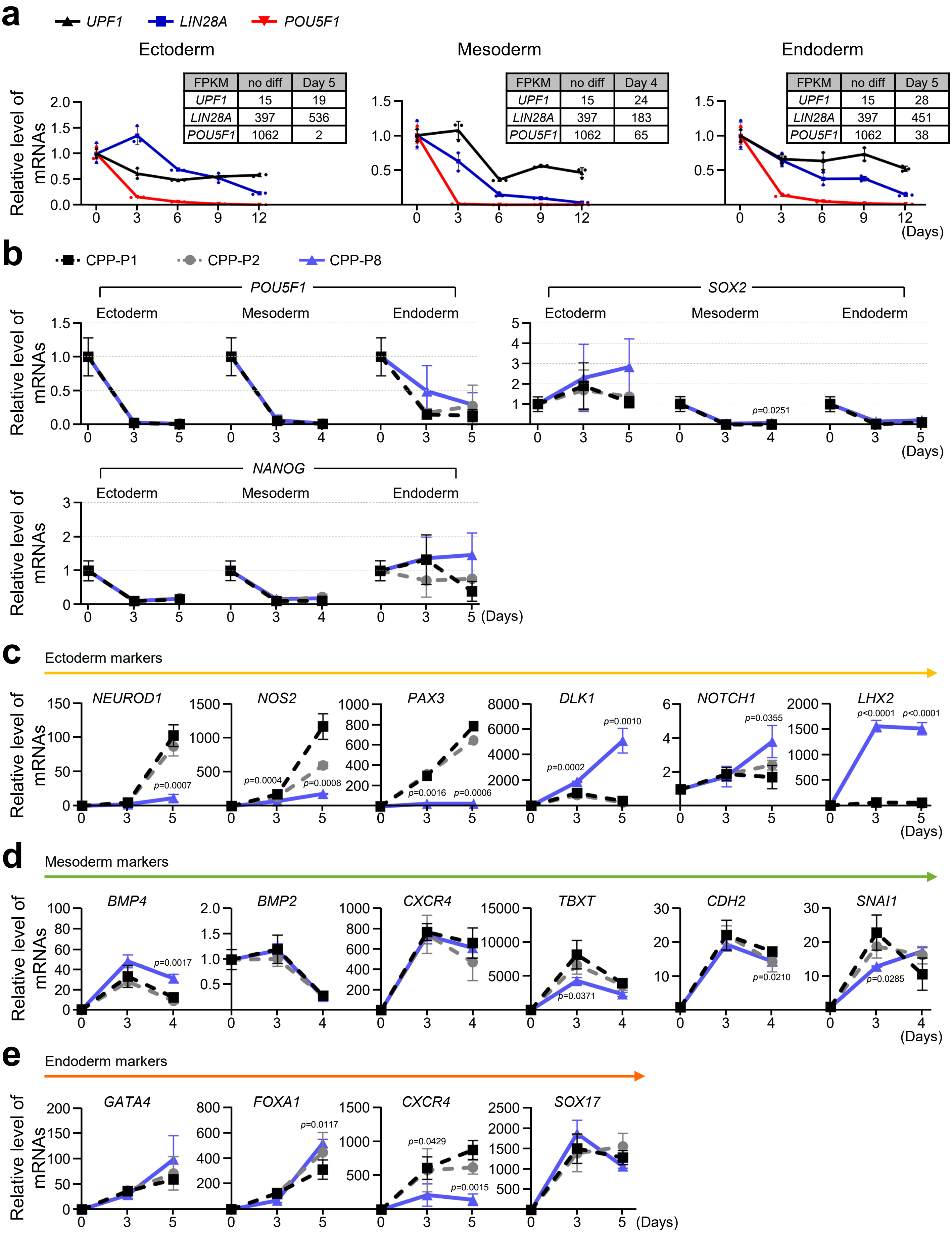

a
UPF1
LIN28A
POU5F1
Ectoderm
Mesoderm
Endoderm
FPKM | no diff | Day 5
UPF1 | 15 | 19
LIN28A | 397 | 536
POU5F1 | 1062 | 2
FPKM | no diff | Day 4
UPF1 | 15 | 24
LIN28A | 397 | 183
POU5F1 | 1062 | 65
FPKM | no diff | Day 5
UPF1 | 15 | 28
LIN28A | 397 | 451
POU5F1 | 1062 | 38
Relative level of mRNAs
(Days)
b
CPP-P1
CPP-P2
CPP-P8
POU5F1
SOX2
NANOG
Ectoderm
Mesoderm
Endoderm
p=0.0251
Relative level of mRNAs
(Days)
c
Ectoderm markers
NEUROD1
NOS2
PAX3
DLK1
NOTCH1
LHX2
p=0.0007
p=0.0004
p=0.0008
p=0.0016
p=0.0006
p=0.0002
p=0.0010
p=0.0355
p<0.0001
p<0.0001
Relative level of mRNAs
(Days)
d
Mesoderm markers
BMP4
BMP2
CXCR4
TBXT
CDH2
SNAI1
p=0.0017
p=0.0371
p=0.0210
p=0.0285
Relative level of mRNAs
(Days)
e
Endoderm markers
GATA4
FOXA1
CXCR4
SOX17
p=0.0117
p=0.0429
p=0.0015
Relative level of mRNAs
(Days)

**f**

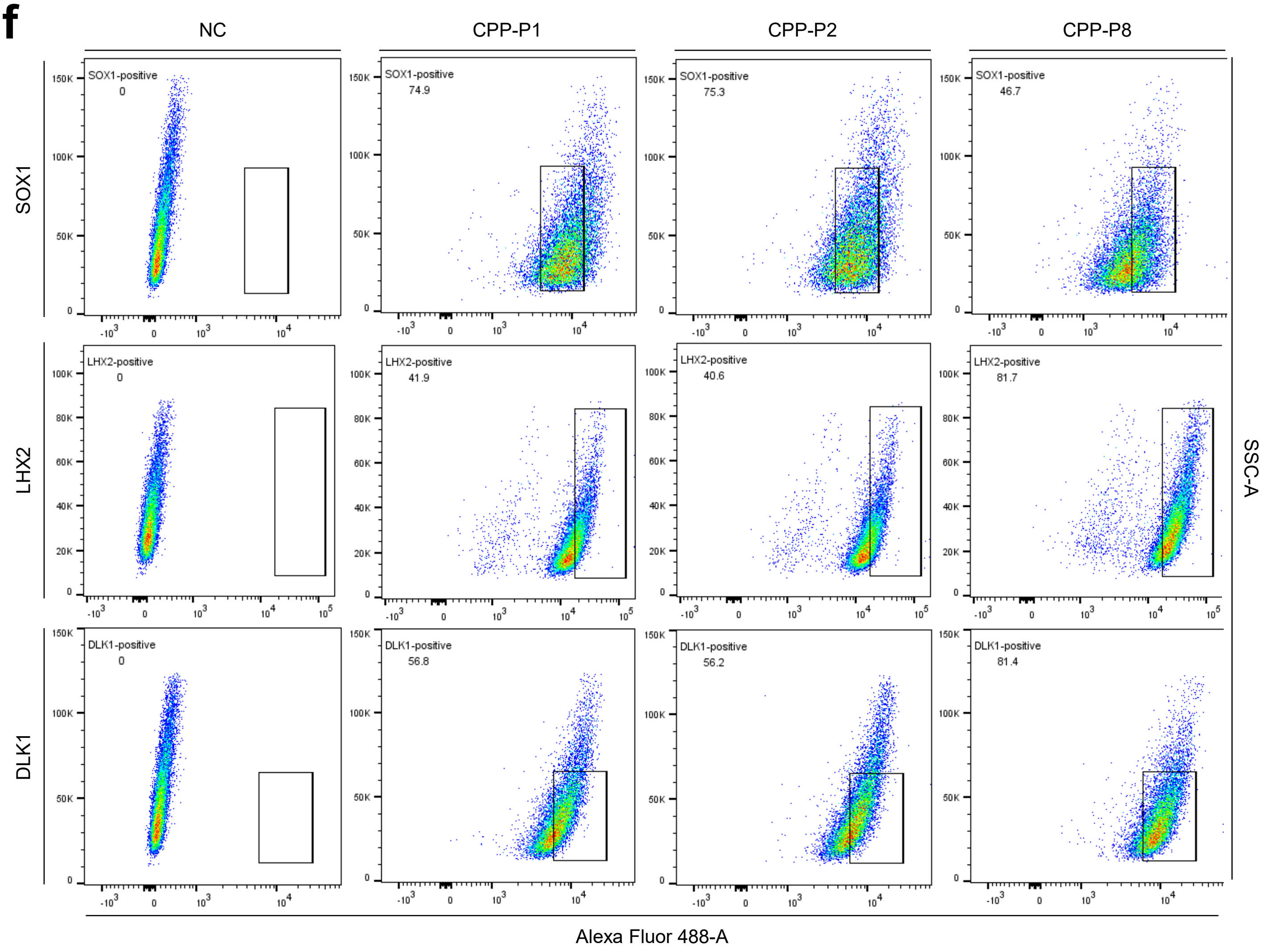


**Supplementary Fig. 7 a** Levels of UPF1, LIN28A, and POU5F1 in hPSCs were assessed using RT-qPCR during each germ layer differentiation for 12 days. FPKM of each gene was presented from RNA-seq analysis. **b**–**e** hPSCs were treated with the indicated peptide during each germ layer differentiation. Relative amounts of pluripotency markers (*POU5F1*, *SOX2*, and *NANOG*) and three germ layers markers were measured using RT-qPCR. mRNA levels were normalised to those of *U6* snRNA in (**a**–**e**). **f** (Corresponding to Fig. 7b) Flow cytometry analysis was performed to detect the levels of OCT4 and NANOG in CPP-P1 or -P8-treated CHA15 cells. The primary antibody that detected the cellular proteins was labelled with the Alexa Fluor 488-conjugated antibody. The relative positive cells were indicated. An unpaired Student's t-test was used for **a**–**e**. Data are presented as mean values ± SEM. All statistical test used were two-sided. The minimum number of independent biological replicate experiments was **a**–**f** $n = 3$. The experiments were conducted three times, each iteration producing consistent results.

**Reference**

1. Heo, I. *et al.* TUT4 in concert with Lin28 suppresses microRNA biogenesis through pre-microRNA uridylation. *Cell* **138**, 696-708 (2009).
