# Supplementary material for: Role of UPF1-LIN28A interaction during early differentiation of pluripotent stem cells
Source: Nat Commun. 2024 Jan 2;15:158. doi: 10.1038/s41467-023-44600-5 (PMC10762078; doi:10.1038/s41467-023-44600-5)
Supplement: Supplementary file 3 — Description of Additional Supplementary Files [file 41467_2023_44600_MOESM3_ESM.pdf]

Description of Additional Supplementary Files

File Name: Supplementary Data 1
Description: BLASTp results of CPP conjugated P2 and P8.

File Name: Supplementary Data 2
Description: The differentially expressed genes commonly regulated by UPF1 knockdown, LIN28A knockdown, and CPP-P8 treatment in Fig. 6b.

File Name: Supplementary Data 3
Description: The complete Gene Ontology (GO) list corresponding to Fig. 6b and 6d.

File Name: Supplementary Data 4
Description: A comprehensive list of primer sequences utilized in the construction of plasmid DNAs.

File Name: Supplementary Data 5
Description: A comprehensive list of siRNA sequences used in knockdown experiments.

File Name: Supplementary Data 6
Description: A comprehensive list of primer sequences used for qPCR and miRNA reverse transcription (RT).
