# Supplementary material for: Role of UPF1-LIN28A interaction during early differentiation of pluripotent stem cells
Source: Nat Commun. 2024 Jan 2;15:158. doi: 10.1038/s41467-023-44600-5 (PMC10762078; doi:10.1038/s41467-023-44600-5)
Supplement: Supplementary file 10 — Reporting Summary [file 41467_2023_44600_MOESM10_ESM.pdf]

nature portfolio

Corresponding author(s): DBPR, NCOMMS-23-06651-T

Last updated by author(s): Nov 6, 2023

# Reporting Summary

Nature Portfolio wishes to improve the reproducibility of the work that we publish. This form provides structure for consistency and transparency in reporting. For further information on Nature Portfolio policies, see our Editorial Policies and the Editorial Policy Checklist.

## Statistics

For all statistical analyses, confirm that the following items are present in the figure legend, table legend, main text, or Methods section.

| n/a | Confirmed | |
|---|---|---|
| ☐ | ☒ | The exact sample size (*n*) for each experimental group/condition, given as a discrete number and unit of measurement |
| ☐ | ☒ | A statement on whether measurements were taken from distinct samples or whether the same sample was measured repeatedly |
| ☐ | ☒ | The statistical test(s) used AND whether they are one- or two-sided<br>*Only common tests should be described solely by name; describe more complex techniques in the Methods section.* |
| ☒ | ☐ | A description of all covariates tested |
| ☐ | ☒ | A description of any assumptions or corrections, such as tests of normality and adjustment for multiple comparisons |
| ☒ | ☐ | A full description of the statistical parameters including central tendency (e.g. means) or other basic estimates (e.g. regression coefficient) AND variation (e.g. standard deviation) or associated estimates of uncertainty (e.g. confidence intervals) |
| ☒ | ☐ | For null hypothesis testing, the test statistic (e.g. *F*, *t*, *r*) with confidence intervals, effect sizes, degrees of freedom and *P* value noted<br>*Give P values as exact values whenever suitable.* |
| ☒ | ☐ | For Bayesian analysis, information on the choice of priors and Markov chain Monte Carlo settings |
| ☒ | ☐ | For hierarchical and complex designs, identification of the appropriate level for tests and full reporting of outcomes |
| ☒ | ☐ | Estimates of effect sizes (e.g. Cohen's *d*, Pearson's *r*), indicating how they were calculated |

*Our web collection on statistics for biologists contains articles on many of the points above.*

## Software and code

Policy information about availability of computer code

| | |
|---|---|
| Data collection | No software was use for data collection. |
| Data analysis | For RNA sequencing data, we pre-processed the raw reads from the sequencer to remove low-quality reads and adapter sequences before analysis and aligned the processed reads to Homo sapiens (GRCh37) using HISAT v2.1.051. HISAT utilises two indices for alignment (a global whole-genome index and tens of thousands of small local indexes). These two types of indices are constructed using the same Burrows-Wheeler transform (BWT)/graph FM index (GFM) as Bowtie2. Transcript assembly was processed using StringTie v1.3.4d.<br>Based on this result, the expression abundance of transcripts and genes was calculated as read count or Fragments Per Kilobase of exon per million fragments mapped (FPKM) value per sample. Differentially expressed genes (DEGs) were analysed by the ratio of FPKM or using DESeq2 with read counts. Log2 fold-change value of the genes was converted into cumulative frequency curve using the R function, 'ecdf' v4.0.5. Random genes were selected using the R function, 'Sample' v4.0.5. Gene functional classification and Gene ontology (GO) were performed using g:Profiler. Sankey diagram and dot plots for GO analysis were plotted by https://www.bioinformatics.com.cn/srplot, an online platform for data analysis and visualization.<br><br>For SPR data, primary data were collected using iMSPR measurement tool and data analysis was performed using TraceDrawer v1.9.2. |

For manuscripts utilizing custom algorithms or software that are central to the research but not yet described in published literature, software must be made available to editors and reviewers. We strongly encourage code deposition in a community repository (e.g. GitHub). See the Nature Portfolio guidelines for submitting code & software for further information.

## Data

Policy information about availability of data

All manuscripts must include a data availability statement. This statement should provide the following information, where applicable:

- Accession codes, unique identifiers, or web links for publicly available datasets
- A description of any restrictions on data availability
- For clinical datasets or third party data, please ensure that the statement adheres to our policy

Raw RNA-seq data have been deposited in the NCBI Gene Expression Omnibus (GEO; https://www.ncbi.nlm.nih.gov/geo/) under accession number GSE224358.

## Human research participants

Policy information about studies involving human research participants and Sex and Gender in Research.

| | |
|---|---|
| Reporting on sex and gender | Not applicable. |
| Population characteristics | Not applicable. |
| Recruitment | Not applicable. |
| Ethics oversight | Not applicable. |

Note that full information on the approval of the study protocol must also be provided in the manuscript.

# Field-specific reporting

Please select the one below that is the best fit for your research. If you are not sure, read the appropriate sections before making your selection.

[x] Life sciences [ ] Behavioural & social sciences [ ] Ecological, evolutionary & environmental sciences

For a reference copy of the document with all sections, see nature.com/documents/nr-reporting-summary-flat.pdf

# Life sciences study design

All studies must disclose on these points even when the disclosure is negative.

| | |
|---|---|
| Sample size | At least three samples were analyzed. Although there is a clear difference, if a statistical significance is marginal, additional samples were performed. |
| Data exclusions | No data exclusion. |
| Replication | All attempt replications were successful. |
| Randomization | Not applicable. |
| Blinding | Not applicable. |

# Reporting for specific materials, systems and methods

We require information from authors about some types of materials, experimental systems and methods used in many studies. Here, indicate whether each material, system or method listed is relevant to your study. If you are not sure if a list item applies to your research, read the appropriate section before selecting a response.

## Materials & experimental systems

| n/a | Involved in the study |
|---|---|
| ☐ | ☒ Antibodies |
| ☐ | ☒ Eukaryotic cell lines |
| ☒ | ☐ Palaeontology and archaeology |
| ☒ | ☐ Animals and other organisms |
| ☒ | ☐ Clinical data |
| ☒ | ☐ Dual use research of concern |

## Methods

| n/a | Involved in the study |
|---|---|
| ☒ | ☐ ChIP-seq |
| ☐ | ☒ Flow cytometry |
| ☒ | ☐ MRI-based neuroimaging |

# Antibodies

Antibodies used

#protein (Vendor, Cat number, RRID)
FLAG (GenScript, Piscataway, NJ, USA, A00187-100, AB_1720813), UPF1 (Cell Signaling Technology, Danvers, MA, USA, 12040, AB_2797806), UPF2 (Cell Signaling Technology, Danvers, MA, USA, 11875, AB_2797752), eIF4E (Cell Signaling Technology, Danvers, MA, USA, 2067, AB_2097675), β-actin (Sigma-Aldrich, St. Louis, MO, USA, A2228, AB_476697), GST (Cytiva, Marlborough, MA, USA, 27-4577-01, AB_771432), MYC (Calbiochem, La Jolla, CA, USA, OP10, AB_564473), Calnexin (Cell Signaling Technology, Danvers, MA, USA, 2679, AB_2228381), Lin28A (rabbit, Cell Signaling Technology, Danvers, MA, USA, 3978, AB_2297060), Lin28A (mouse, Cell Signaling Technology, Danvers, MA, USA, 5930, AB_1903976), phosphorylated UPF1 (from Dr.Yoonki Kim of Korea University, pSer1096), phosphorylated UPF1 (p-Thr28, ImmuQuest, Durham, UK, IQ653), OCT4 (Santa Cruz, Dallas, TX, USA, sc-5279, AB_628051), NANOG (Cell Signaling Technology, Danvers, MA, USA, 4903, AB_10559205), DLK1 (GeneTex, Irvine, CA, USA, GTX60511, AB_2927649), SOX1 (Cell Signaling Technology, Danvers, MA, USA, 4194, AB_1904140), and LHX2 (GeneTex, Irvine, CA, USA, GTX129241, AB_2783558)

Validation

All antibodies except pSer1096 UPF1 antibody were purchased from the commercial companies with catalog numbers listed above.

# Eukaryotic cell lines

Policy information about cell lines and Sex and Gender in Research

Cell line source(s)

HeLa cells (KCLB, 10002), 293T cells (KCLB, 21573), PA-1 cells (ATCC, CRL-1572), CHA-hES15 (CVCL, 9741), H9 cells (WiCell) , and Pro2(iPSCs were kindly gifted by Dr. Kwang-Soo Kim of Harvard University)

Authentication

Cell line was purchased from Korean Cell Line Bank (KCLB) and ATCC. CHA-hES15 and Pro2 cells were gifted from CHA university and Harvard university, respectively, as described in the Methods section

Mycoplasma contamination

All the cell lines were routinely tested and confirmed that there is no contamination.

Commonly misidentified lines
(See ICLAC register)

*Name any commonly misidentified cell lines used in the study and provide a rationale for their use.*

# Flow Cytometry

## Plots

Confirm that:

☒ The axis labels state the marker and fluorochrome used (e.g. CD4-FITC).

☒ The axis scales are clearly visible. Include numbers along axes only for bottom left plot of group (a 'group' is an analysis of identical markers).

☒ All plots are contour plots with outliers or pseudocolor plots.

☒ A numerical value for number of cells or percentage (with statistics) is provided.

## Methodology

Sample preparation

To evaluate the relative cell number expressing the indicated proteins, CHA15 cells with the indicated CPP-conjugated peptide were fixed with BD Cytofix Fixation Buffer (BD Biosciences Pharmingen, 554655) followed by permeabilization with 0.1% BSA/PBS solution supplemented with 10% NGS and 0.3% Triton X-100 for 1 h at 4°C. After washing, cells were incubated with the primary antibodies (OCT4 1:1000, NANOG 1:1000, DLK1 1:1000, SOX2 1:1000, LHX2 1:1000) and then, secondary antibody, Alexa Fluor 488 (1:1000), was applied.

Instrument

The positive cells were determined using flow cytometry (FACSCanto, BD Pharmingen)

Software

Data was analysed using the Flow Jo-v10 software program.

Cell population abundance

The proportion of living cells was always higher than 95%.

Gating strategy

Cells were selected based on their forward scatter (FSC-A) and side scatter (SSC-A) properties. Subsequently, cell debris and

non-viable cells were excluded from the analysis by applying size and complexity criteria. To discriminate doublets, cells were further gated using a FSC-A vs SSC-A plot. Negative controls consisted of unstained cells. In the case of cells subjected to antibody incubation, the expression levels were quantified based on the fluorescence intensity of Alexa 488.

☐ Tick this box to confirm that a figure exemplifying the gating strategy is provided in the Supplementary Information.
